# Replication of Crohn’s Disease Mucosal *E. coli* Isolates inside Macrophages Correlates with Resistance to Superoxide and Is Dependent on Macrophage NF-kappa B Activation

**DOI:** 10.3390/pathogens8020074

**Published:** 2019-06-08

**Authors:** Ahmed Tawfik, Paul Knight, Carrie A. Duckworth, D. Mark Pritchard, Jonathan M. Rhodes, Barry J. Campbell

**Affiliations:** 1 Gastroenterology Research Unit, Department of Cellular & Molecular Physiology, Institute of Translational Medicine, University of Liverpool, Liverpool L69 3GE, UK; ahmedtawfik13@yahoo.com (A.T.); paul.knight@mft.nhs.uk (P.K.); carried@liv.ac.uk (C.A.D.); dmpritch@liv.ac.uk (D.M.P.); rhodesjm@liv.ac.uk (J.M.R.); 2 Gastroenterology Department, Beaumont Hospital, Dublin 9, Ireland; 3 Gastroenterology Department, University Hospital of South Manchester, Wythenshawe M23 9LT, UK

**Keywords:** *Escherichia coli*, Crohn’s disease, macrophage, phagolysosome, superoxide, hydrocortisone, Nuclear factor kappa B

## Abstract

Mucosa-associated *Escherichia coli* are increased in Crohn’s disease (CD) and colorectal cancer (CRC). CD isolates replicate within macrophages but the specificity of this effect for CD and its mechanism are unclear. Gentamicin exclusion assay was used to assess *E. coli* replication within J774.A1 murine macrophages. *E. coli* growth was assessed following acid, low-nutrient, nitrosative, oxidative and superoxide stress, mimicking the phagolysosome. Twelve of 16 CD *E. coli* isolates replicated >2-fold within J774.A1 macrophages; likewise for isolates from 6/7 urinary tract infection (UTI), 8/9 from healthy subjects, compared with 2/6 ulcerative colitis, 2/7 colorectal cancer and 0/3 laboratory strains. CD mucosal *E. coli* were tolerant of acidic, low-nutrient, nitrosative and oxidative stress. Replication within macrophages correlated strongly with tolerance to superoxide stress (rho = 0.44, *p* = 0.0009). Exemplar CD *E. coli* HM605 and LF82 were unable to survive within *Nfκb1^-/-^* murine bone marrow-derived macrophages. In keeping with this, pre-incubation of macrophages with hydrocortisone (0.6 µM for 24 h) caused 70.49 ± 12.11% inhibition of intra-macrophage replication. Thus, CD mucosal *E. coli* commonly replicate inside macrophages, but so do some UTI and healthy subject strains. Replication correlates with resistance to superoxide and is highly dependent on macrophage NF-κB signalling. This may therefore be a good therapeutic target.

## 1. Introduction

Mucosa-associated *E. coli* have been found in increased numbers on the ileal and colonic mucosae, including the inner adherent mucus layer, of patients with Crohn’s disease (CD) [1,2,3,4,5,6] or colorectal cancer (CRC) [3,4,5,6,7], and to a lesser extent, patients with ulcerative colitis (UC) [8,9]. A high proportion of CD mucosal *E. coli* strains adhere to, and invade, intestinal epithelial cell-lines Caco-2 and Int-407, and induce release of pro-inflammatory cytokines [2,3,4,5,6,10]; however, it has been noted that the level of invasion into epithelial cell lines is strongly dependent on the cell line chosen for experimental study [3]. In vitro studies using the paradigm CD *E. coli* strains from the ileum (LF82) [1] and from the colon (HM605) [3] showed that they also possess the ability to replicate within murine and human monocyte-derived macrophages (HMDM) [11,12,13,14] and to induce granuloma formation, a hallmark of Crohn’s disease [15]. Electron microscopy confirmed replication of CD strains LF82 and HM605 inside phagolysosomes within macrophages [11,12,13,14] and this replication was shown to favour the presence of an acidic environment [12]. 

Macrophages play a key role in the host’s immunological and inflammatory response [16]. Activated macrophages are classified into classically activated macrophages (M_1_-macrophages), which are immune effectors against pathogenic bacteria and associated with marked production of lymphokines, and alternatively activated macrophages (M_2_-macrophages) having a variety of functions, including immune regulation, tissue repair and wound healing [16,17]. Genome-wide association studies (GWAS) have now identified more than 200 IBD-associated loci [18,19]. In CD, particularly, these include gene polymorphisms relating to innate immune system functions such as pathogen recognition (nucleotide-binding oligomerisation domain-containing-2/Caspase-recruitment domain 15 (*NOD2*/*CARD15*) and interleukin 23 receptor (*IL23R*)) and autophagy (immunity-related GTPase M (*IRGM*) and autophagy-related 16-like 1 (*ATG16L1*)), which are highly relevant to killing of bacteria within macrophages [19].

In vivo studies in CD patients demonstrated a defect in neutrophil recruitment along with an abnormal production of cytokines following either acute trauma to the rectum or subcutaneous injection of heat-killed *E. coli* [20,21,22]. However, ex vivo studies reported normal chemotaxis of CD neutrophils [23,24]. We have previously reported no significant differences in killing of CD mucosal *E. coli* HM605, *E. coli* K-12 and *Staphylococcus aureus* Oxford strain between CD and healthy control peripheral blood monocyte-derived macrophages; moreover, active CD macrophages also showed an equivalent ability to induce neutrophil chemotaxis in vitro relative to unaffected controls [25]. No consistent abnormality in phagocytosis and respiratory burst function of MDM obtained from CD patients has been described to date [26]. 

Bacteria phagocytosed by macrophages are surrounded by a double membrane to form internal vesicles termed phagosomes. These phagosomes fuse with lysosomes to form mature phagolysosomes, in which their contents are degraded by reactive oxygen species (ROS) and proteolytic enzymes [27,28]. ROS are chemically reactive molecules containing free oxygen radicals, including superoxide anions (O_2_^-^), hydrogen peroxide (H_2_O_2_), and hydroxyl radicals (OH^-^) produced within the intracellular compartment under aerobic conditions through the activation of multicomponent NADPH oxidase. ROS are cytotoxic and are essential in the microbicidal process of macrophages [29,30]. CD mucosal *E. coli* strains, during the later phase of murine macrophage infection, induce chronic activation of NF-κB, which correlates with increased tumour necrosis factor (TNF) secretion [31]. CD mucosal *E. coli* replication within macrophages has been reported to be dependent on TNF secretion [11,32]. 

The combination of adherence to, and invasion of, epithelial cell lines plus the ability to replicate within macrophages led to the phenotypic designation of CD mucosa-associated *E. coli* as Adherent, Invasive *E. coli* (AIEC) [10]. However, no genotype has been defined for AIEC [33], although CD isolates (and CRC isolates) more commonly possess key virulence genes that drive common phenotypic/pathogenic actions, including epithelial cell adherence and invasion, entry via microfold (M) cells of the follicle-associated epithelium, enhanced angiogenic potential and genotoxicity [34,35,36,37,38]. They are possibly best defined as pathobionts—organisms with the potential for causing disease that likely live as symbionts under circumstances of normal gut health. 

Here we compare the replication within macrophages of CD, UC and CRC mucosally associated *E. coli* strains, *E. coli* strains from patients with urinary tract infection (UTI), *E. coli* strains from healthy individuals with no evidence of intestinal inflammation, and laboratory *E. coli* strains. We have also assessed their tolerance of stress conditions characteristic of the macrophage phagolysosomes and the role of the classical NF-κB pathway activation in this process.

## 2. Results

### 2.1. Ability to Survive and Replicate within Macrophages Is Not Confined Specifically to CD Mucosal *E. coli* Strains 

**Figure 1:**
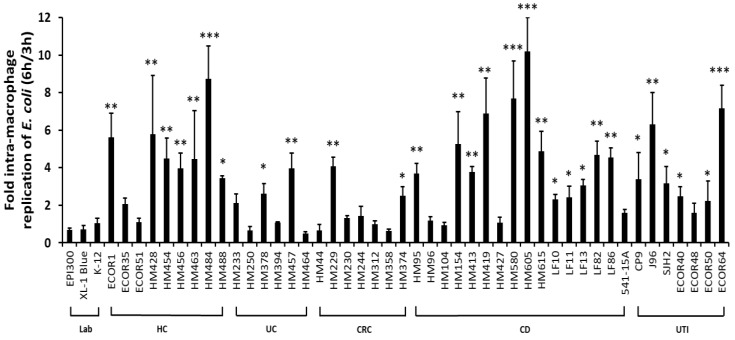
Crohn’s disease mucosa-associated *E. coli* isolates show significant intra-macrophage replication. Replication of 48 *E. coli* (*n* = 16 CD, *n* = 7 CRC, *n* = 6 UC, *n* = 9 HC, *n* = 7 UTI and *n* = 3 laboratory strains) was assessed by overnight bacterial culture of J774.A1 murine macrophage cell lysates following a gentamicin protection assay. Data are expressed as fold change [mean ± SD] of recovered intra-macrophage bacteria 6 h post-infection compared to the number of viable colony-forming units (cfu) obtained immediately after a 1-h gentamicin treatment step (i.e., 3 h post-infection). *N* = 3 independent experiments, *n* = 3 replicates, excepting for *E. coli* LF82 and EPI300 (*N* = 9, *n* = 3), and HM605 (*N* = 6, *n* = 3). Significant differences are indicated as follows: *p < 0.05, **p < 0.01 and ***p < 0.001; ANOVA with Dunnett’s post hoc test compared to control (non-replicating laboratory *E. coli* EPI300).

CD mucosal *E. coli* strains more commonly showed an ability to replicate within J774-A1 murine macrophages with 12 out of 16 CD strains examined showing >2-fold replication (Figure 1); *p* = 0.036 versus 0/3 laboratory *E. coli* studied; two-sided Fisher’s exact test. Mean fold replication for all isolates across the CD group (± SD) was significantly higher at 4.01-fold (± 2.60) versus laboratory *E. coli* studied [0.82-fold (± 0.29)]; (*N* = 3–9 experiments, *n* = 3 replicates; *p* < 0.01, ANOVA). The paradigm ileal CD AIEC LF82, and the colonic CD AIEC HM605, both showed significant intra-macrophage replication (LF82, 4.67 ± 0.73-fold (*N* = 9, *n* = 3) and HM605 10.18 ± 1.82-fold (*N* = 6, *n* = 3); both *p* < 0.001) compared to non-pathogenic *E. coli* EPI300 (0.67 ± 0.11-fold; *N* = 9, *n* = 3). Only 2/6 UC strains (HM233 and HM457) and 2/7 CRC strains (HM229 and HM374) were seen to replicate to a level >2-fold at 6 h/3 h (Figure 1). Overall mean fold intra-macrophage replication the group of *E. coli* isolates tested from UC and CRC patients were 1.48 ± 1.37-fold and 1.46 ± 1.24-fold replication, respectively. Of note, significant replication within macrophages was also observed for some non-CD isolates (see Figure 1). This included 6/7 *E. coli* strains obtained from patients with urinary tract infection (UTI), *p* = 0.03 (Fishers exact test); with mean fold replication overall being 3.76-fold (± 2.13) higher across all UTI isolates tested, *p* = 0.0098 ANOVA compared to laboratory *E. coli* studied. Significant survival and replication was seen for two particular UTI isolates, one from a patient with acute cystitis, ECOR64 (7.15 ± 1.24-fold; *p* < 0.001, ANOVA; *N* = 3, *n* = 3) and the second the uropathogenic *E. coli* (UPEC) J96 (6.30 ± 1.70-fold; *p* < 0.001), with all other UTI-associated *E. coli* showing some trend for intra-macrophage replication bar ECOR48, 1.59 ± 0.52-fold compared to laboratory strain EPI300. Interestingly, eight out of nine *E. coli* strains obtained from colonic mucosal samples from healthy individuals (i.e., with no evidence of intestinal inflammation) also showed significant replication; this included all colonic mucosa-associated HM strains tested, ECOR35 (a group D strain) and ECOR1 (a group A strain) from healthy individuals, the latter replicating significantly at 5.62 ± 1.28-fold within J774.A1 macrophages (*p* < 0.01, ANOVA) compared with laboratory strain EPI300. 

### 2.2. Crohn’s Disease Mucosa-Associated *E. coli* Strains Show Ability to Tolerate Stress Conditions that Mimic the Phagolysosome Environment, in Particular Superoxide Stress

**Figure 2:**
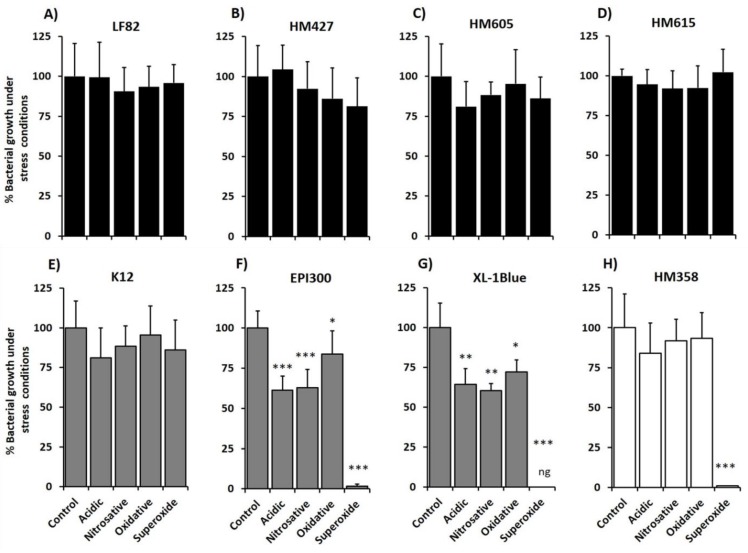
Mucosa-associated *E. coli* strains all tolerate acidic, nitrosative and oxidative stress but vary considerably in their ability to tolerate superoxide stress that mimics the phagolysosome environment. CD mucosa-associated *E. coli* strains LF82 (**A**), HM427 (**B**), HM605 (**C**) and HM615 (**D**) (black bars) showed significant ability to tolerate acidic stress (LB agar containing 100 mM morpholine ethanesulphonic acid [MES], pH 5.0), nitrosative stress (LB agar containing 100 mM MES pH 5.0 and 1mM NaNO_2_), oxidative stress (LB agar containing 1 mM H_2_O_2_, pH 7.0) and superoxide stress (LB agar containing 1 mM methyl viologen, pH 7.0), compared to growth on reference LB agar pH 7.0. *E. coli* K-12 (E), was also tolerant to all stress conditions. Conversely, laboratory *E. coli* strains EPI300 (**F**) and XL-1Blue (**G**) (grey bars) were intolerant to all studied stress conditions, especially superoxide stress (ng = no growth), and colorectal cancer (CRC) *E. coli* strain HM358 (H) (white bars) was intolerant only to superoxide stress. Significant differences from growth on LB agar pH 7.0; *p < 0.05, **p < 0.01, ***p < 0.001, ANOVA with Dunnett’s post hoc test (*N* = 4 experiments, *n* = 3 replicates).

**Figure 3:**
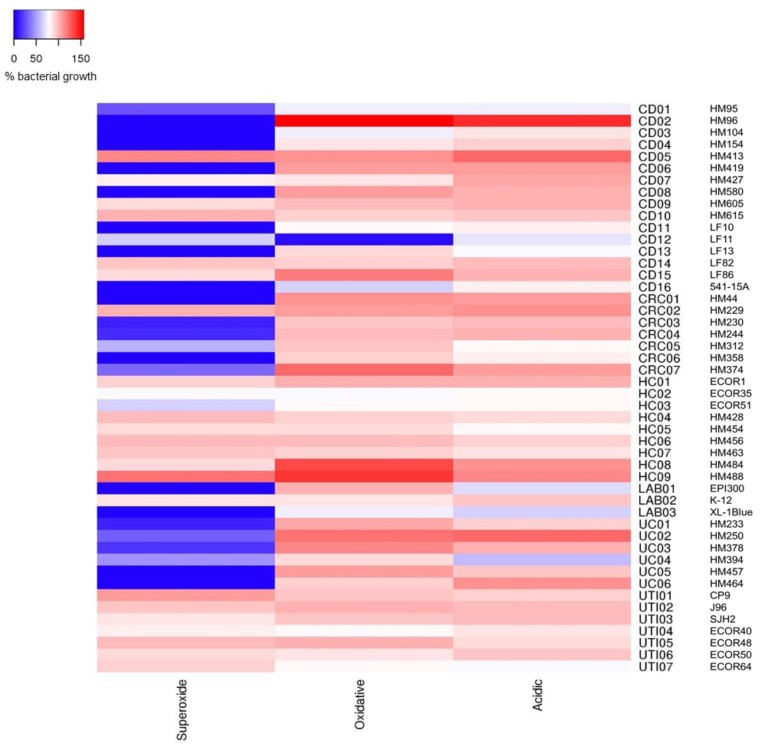
Stress tolerance heatmap of *E. coli* growth on solid media under conditions that mimic the phagolysosome environment. Data expressed as mean % bacterial growth under stress conditions (blue = intolerant, red = high tolerance) compared to growth on reference LB agar pH 7.0 (100%); *N* = 4 experiments, *n* = 3 replicates. Acidic stress (LB agar containing 100 mM morpholine ethanesulphonic acid [MES] pH 5.0); oxidative stress (LB agar containing 1 mM H_2_O_2_ pH 7.0); and superoxide stress (LB agar containing 1 mM methyl viologen pH 7.0). CD, Crohn’s disease; CRC, colorectal cancer; UC, ulcerative colitis; UTI, urinary tract infection; HC, healthy controls; LAB, Laboratory strains.

The growth of adherent, invasive CD *E. coli* strains LF82 and HM605 was not influenced by any of the chemical stress conditions of low pH, high nitrosative, oxidative and high oxidative stress, with no differences in level of growth compared with that seen on reference LB agar, pH 7.0; *N* = 4, *n* = 3 (Figure 2). A similar pattern of stress tolerance was also observed for four other CD mucosa-associated *E. coli* isolates of the 16 tested; LF86, HM413, HM427 and HM615 (see Figure 2 and Figure 3). Of note too, *E. coli* K-12 also tolerated all stress challenge conditions (Figure 2). However, the growth of the two laboratory *E. coli* strains, EPI300 and XL-1Blue, both incapable of survival and replication within macrophages, was significantly suppressed under all stress conditions tested, particularly with superoxide stress (all *p* < 0.05, ANOVA; *N* = 4, *n* = 3). CRC mucosa-associated *E. coli* strain HM358 was seen to tolerate growth on LB agar at pH 5.0, under nitrosative stress and H_2_O_2_-mediated oxidative stress growth conditions but not in an environment of superoxide stress induced by 1 mM methyl viologen (Figure 2). 

Further examination of *E. coli* growth tolerance to acid, oxidative and, in particular, superoxide stress, was undertaken using additional strains obtained from healthy individuals and various patient groups. Those isolates showing >2-fold intra-macrophage survival within these groups were commonly found to be tolerant of acid, oxidative and superoxide stress. This was the case for all six UTI strains with >2-fold intra-macrophage replication, where the % mean growth was 78‒99% in acidic conditions, 81‒103% in H_2_O_2_, induced oxidative stress and 84‒107% in superoxide stress. Even the sole UTI isolate showing <2-fold replication, ECOR48, was tolerant to all three growth stress conditions (see Figure 3). Eight of nine isolates from healthy subject controls (i.e., without bowel inflammation) showing >2-fold intra-macrophage survival, also showed significant tolerance to all three stress conditions; this included irritable bowel syndrome patient isolates (HM484 and HM488), and isolates from patients with sporadic polyps (HM428, HM454, HM456) or haemorrhoids (HM463). The two intra-macrophage replicating UC strains (HM233 and HM457) were only observed to be growth tolerant to acidic and oxidative stress. Of the two CRC strains seen to replicate >2-fold, only HM229 was tolerant to all three stress conditions, whereas HM374 could not tolerate superoxide stress (see Figure 3).

Overall, significant tolerance to superoxide stress was observed between *E. coli* from different sources (*p* = 0.0039; Kruskal‒Wallis ANOVA; *N* = 4 experiments, *n* = 3 replicates), particularly UTI-associated *E. coli* (7/7), strains from CD patients (6/16) and healthy controls (7/9) (see Figure 3). Laboratory *E. coli*, excepting *E. coli* K-12, were intolerant to superoxide stress. All UC strains (*n* = 6) were intolerant to superoxide. Likewise, CRC-mucosa associated *E. coli* strains tested (*n* = 7) whilst able to tolerate growth at pH 5.0 and oxidative stress induced by H_2_O_2_, were intolerant to superoxide stress, with the sole exception being HM229. No differences were seen in tolerance between *E. coli* from different sources with respect to acidic nor oxidative stress (*p* = 0.3852 and *p* = 0.1224 respectively). One notable exception, ileal CD isolate LF11, was extremely sensitive to H_2_O_2_-induced oxidative stress (Figure 3).

### 2.3. Correlation of *E. coli* Tolerance to Methyl Viologen Induced Superoxide Stress with Ability to Replicate within Macrophages

**Figure 4:**
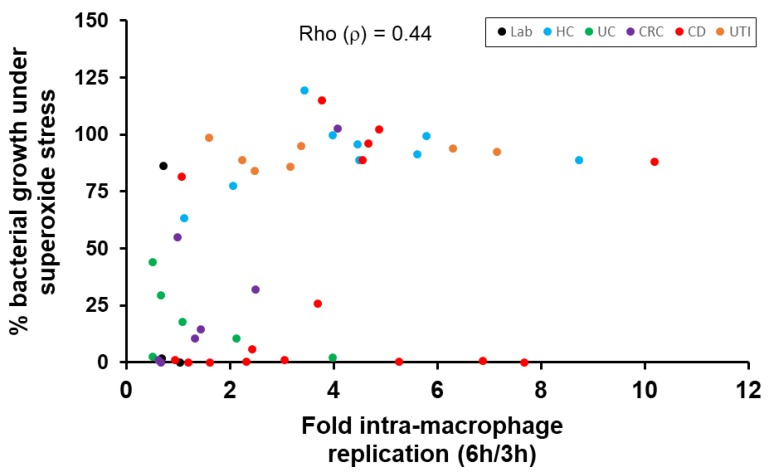
Correlation between growth of *E. coli* strains under superoxide stress conditions and their ability to replicate within macrophages. Spearman’s rank correlation shows a monotonic direct relationship between growth of 48 *E. coli*; *n* = 16 CD [Crohn’s disease]—red circles, *n* = 7 CRC [colorectal cancer] —purple, *n* = 6 UC [ulcerative colitis [green], *n* = 9 HC [healthy control individuals] —light blue, *n* = 7 UTI [urinary tract infection] – orange circles and *n* = 3 Lab [laboratory] strains—black) on solid media under superoxide stress (LB agar containing 1 mM methyl viologen, pH 7.0) and their ability to survive and replicate within J774.A1 murine macrophages. Coefficient Rho (ρ) = 0.44; two-sided *p* = 0.0009.

A marked correlation was observed between replication of 48 *E. coli* strains (*n* = 16 CD, *n* = 7 CRC, *n* = 6 UC, *n* = 9 HC, *n* = 7 UTI and *n* = 3 laboratory strains) inside macrophages and their growth on LB agar under methyl viologen-induced superoxide stress (ρ = 0.44 [95% CI, 0.47 to 0.86], two-sided *p* = 0.0009, Spearman’s rank correlation coefficient) (Figure 4). 

### 2.4. Crohn’s Disease *E. coli* Isolates Are Able to Tolerate a Low-Nutrient, Acidic Environment

**Figure 5:**
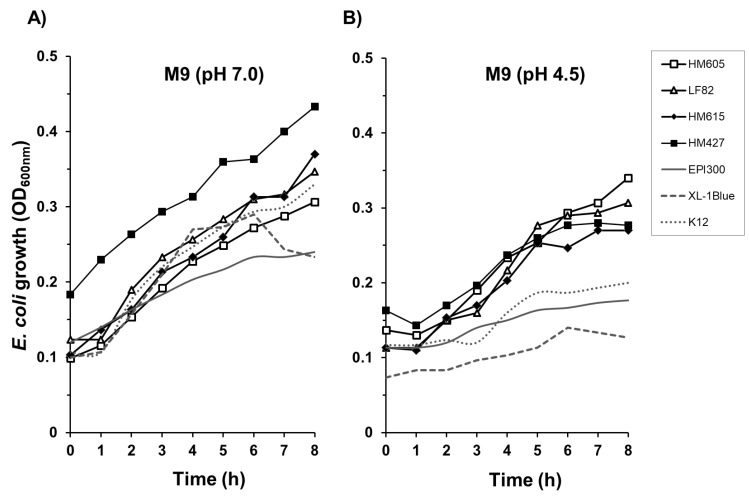
Crohn’s disease mucosa-associated *E. coli* strains are able to grow within low-nutrient, acidic conditions characteristic of the macrophage phagolysosome environment. Comparison of CD mucosa-associated *E. coli* strains to non-intramacrophage replicating laboratory *E. coli* strains (EPI300, XL-1Blue and K-12) in low-nutrient culture medium (M9 minimal salts microbial growth medium supplemented with 0.1% w/v casamino acids, 100 mM Bis-Tris, 0.16% v/v glycerol and 10 μM magnesium chloride) at pH 7.0 (**A**) and pH 4.5 (**B**). All four CD *E. coli* strains showed tolerance over 8 h to low-nutrient M9 media, at pH 4.5. Laboratory *E. coli* strains were unable to grow well at pH 4.5 in M9 minimal media. Lines represent means of triplicate experimental cultures, with *n* = 3 replicates.

Four CD mucosa-associated *E. coli* strains showing tolerance to all chemical stress conditions on solid agar (LF82, HM427, HM605 and HM615) were further examined for their ability to grow in nutrient-limiting conditions encountered within the macrophage phagolysosome. All four isolates showed tolerance over 8 h to low-nutrient M9 minimal culture medium both at pH 7.0 and under acidic conditions, at pH 4.5 (see Figure 5). Laboratory *E. coli* strains K-12, EPI300 and XL-1Blue, which show little or no replication within murine macrophages, were able to grow in minimal M9 medium at pH 7.0 but not at pH 4.5. No bacteria studied were able to grow in low-nutrient M9 minimal media at pH 4.0; *N* = 3, *n* = 3.

### 2.5. Crohn’s Disease Mucosal *E. coli* HM605 and LF82 Replicate inside C57BL/6 Murine Bone Marrow-Derived Macrophages (BMDM), but Are Unable to Survive within Nfκb1-Deficient BMDM

**Figure 6:**
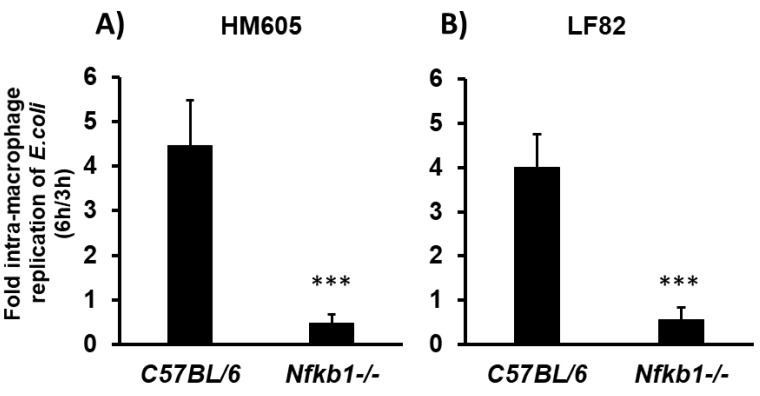
CD mucosa-associated *E. coli* are unable to survive within *Nfκb1*-deficient murine bone-marrow derived macrophages (BMDM). Intra-macrophage replication of paradigm CD adherent, invasive *E. coli* HM605 (**A**) and LF82 (**B**) was significantly reduced within *Nfκb1^-/-^* BMDM compared with those from C57BL/6 mice as determined by gentamicin protection assay. Data are expressed as relative fold change [mean ± SD] of recovered intra-macrophage bacteria 6 h post-infection compared to the number of viable colony-forming units (cfu) obtained immediately after a 1-h gentamicin treatment step (i.e., 3 h post-infection). Data obtained from differentiated BMDMs obtained from bone progenitor cells from four C57BL/6 mice (two male, two female); and four *Nfκb1^-/-^* transgenic mice (one male, three female). Significant differences compared to C57BL/6 BMDMs ***p < 0.001, unpaired t-test.

It has been suggested that CD mucosal adherent, invasive *E. coli* (AIEC) strains can regulate the classical NF-κB signalling pathway to support their survival and persistence within the macrophage phagolysosome, including the prototype CD ileal-mucosa-associated AIEC strain LF82 [31]. We therefore selected this strain and another CD colonic-mucosa-associated AIEC, HM605, and examined for their ability to replicate within wild-type versus *Nfκb1*-deficient bone marrow-derived macrophages (BMDM). Intra-phagolysosome survival and replication of both CD mucosa-associated *E. coli* strains within wild-type C57BL/6 mouse BMDM at 6 h post-infection was >4-fold above that seen at 3 h post-infection (LF82; 4.00 ± 0.75-fold; HM605, 4.47 ± 1.00-fold [mean ± SD])**.** However, both strains were unable to survive inside BMDM derived from *Nfκb1^-/-^* mice (0.47 ± 0.19-fold and 0.56 ± 0.28-fold, respectively, for HM605 and LF82 (both *p* < 0.001 compared to replication within C57BL/6 BMDM, unpaired *t*-test); see Figure 6. Phagocytosis of *E. coli* was not significantly different at 3 h in Nfκb1-deficient versus C57Bl/6 BMDMs (HM605, 41750 ± 16645 vs. 64500 ± 10240; LF82, 27500 ± 5928 vs. 23500 ± 5260 [mean cfu ± SD].

### 2.6. Macrophage Pre-Incubation with the Corticosteroid Hydrocortisone Inhibits Intra-Macrophage Replication of a Crohn’s Disease Mucosa-Associated *E. coli*

**Figure 7:**
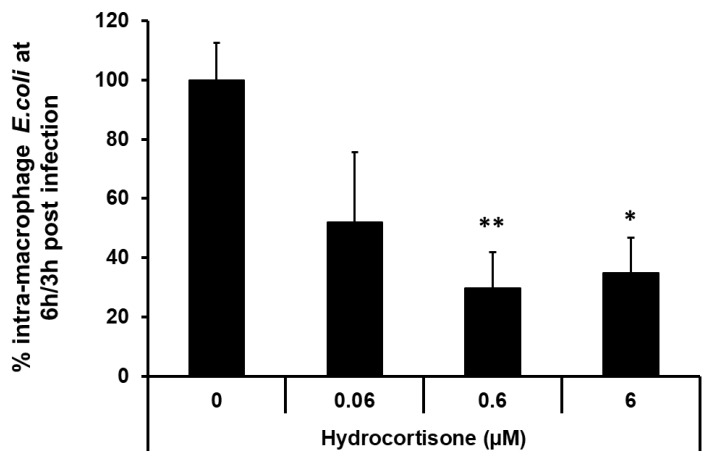
The effect of hydrocortisone on survival and replication of CD mucosa-associated *E. coli* HM605 within macrophages. J774A.1 murine macrophages were pre-treated with hydrocortisone for 24 h prior to inoculation with intra-macrophage replicating CD colonic mucosa-associated *E.coli* HM605. Data are expressed as mean % intra-macrophage bacteria (± SEM) present at 6 h/3 h post-infection, as assessed using a gentamicin protection assay. Significant differences compared to vehicle-treated macrophages, *p < 0.05 and **p < 0.01; Kruskal‒Wallis non-parametric ANOVA: *N* = 4, *n* = 12.

Pre-incubation of macrophages with hydrocortisone causes a dose-dependent decrease in survival of intra-macrophage CD colonic mucosa-associated *E. coli* HM605 in J774A.1 murine macrophages, with maximal effect, 70.49 ± 12.11% (mean ± SEM) inhibition, seen at 0.6 µM (*p* < 0.01, Kruskal‒Wallis ANOVA; *N* = 4 experiments, *n* = 12 replicates); Figure 7. Preliminary experiments showed that the 24 h pre-incubation with hydrocortisone, at highest concentration tested (6 μM), had no significant effects on J774-A1 macrophage cell viability (untreated control 3.27 ± 0.86 versus hydrocortisone 2.56 ± 0.60 [mean ± SEM cells x10^5^; *N* = 3, *n* = 12]) with no significant release of adenylate kinase to culture medium (untreated control 23,700 ± 7510 versus hydrocortisone 20,100 ± 6660 [mean luminescence units ± SEM; *N* = 2, *n* = 6]). Hydrocortisone, at 6 μM, also had no direct effect on *E. coli* growth in culture medium for 6 h in the absence of macrophages, as assessed by enumeration of bacteria cfu following overnight growth on LB agar (Expt 1, control 1.64 ± 0.06 versus hydrocortisone 2.47 ± 0.11; Expt 2, control 3.91 ± 0.12 versus hydrocortisone 5.97 ± 0.69 [mean cfu x10^7^ ± SEM; both *n* = 6]).

## 3. Discussion

The ability of CD mucosal *E. coli* to survive and replicate within the phagolysosome following engulfment by mucosal macrophages confirms previous findings by ourselves [13,14] and others [11,12]. Although colonic *E. coli* strains examined here from UC and CRC patients did not show significant intra-macrophage replication, this phenotype is not specific to CD mucosal strains. UTI-associated strains also appeared to possess this property, with good survival and replication seen in murine macrophages. This confirms previous findings that uropathogenic *E. coli* (UPEC) strains isolated from patients with UTI can survive and replicate within murine macrophages, as well as possessing the ability to replicate within urogenital epithelial cells [39,40]. We also observed that the ability of *E. coli* to replicate within macrophages correlated strongly with an ability to tolerate a superoxide stress environment, as induced by methyl viologen within a growth medium, potentially mimicking the conditions encountered by bacteria inside a macrophage phagolysosome. All UTI-associated *E. coli* strains, including some key UPEC (CP9, J96 and SJH2) tested in this study, were able to tolerate superoxide stress. Key adherent, invasive mucosal *E. coli* from CD patients, including LF82 and HM605, were also tolerant of superoxide stress, although this ability appears to be variable, with other CD strains being intolerant. Of note, *E. coli* K-12, which was not observed to replicate within macrophages, was able to tolerate superoxide stress, and indeed tolerated all growth stress conditions tested here. This strain has previously been reported to tolerate reactive oxygen stress, growing well in LB broth containing 1.5 mM H_2_O_2_ over 24 h [41]; this may perhaps explain its ability to persist within murine J774-A1 macrophages and human peripheral blood monocyte-derived macrophages over 6 h, but not replicate to any significant degree, as was seen for CD mucosa-associated *E. coli* strains [13,14]. 

Overall, our data showed a strong correlation of *E. coli* tolerance to methyl viologen induced superoxide stress at pH 7.0 with the ability to replicate within macrophages. It was not possible to conduct successfully in vitro experiments to further mimic the phagolysosome environment, i.e., superoxide stress at low pH (pH 5.0), as we found that at pH < 6.0, methyl viologen was not able to generate superoxide radicals effectively, as has also been previously described [42]. Thus, mimicking growth conditions with all elements of the phagolysosome environment to monitor CD *E. coli* survival and replication is challenging and may only be achievable in *E. coli*-infected macrophages in culture or in vivo with use of multiphoton microscopy and oxidative, nitrosative and pH sensitive molecular probes, and probes that could measure proteolytic enzyme activities too. 

Tolerance of the acidic, nutrient-limiting environment inside intra-macrophage phagolysosome is important for the survival and replication of key enteric bacteria such as *Salmonella* spp. [43,44] and *Mycobacterium* spp. [45,46], and has also been suggested for the exemplar ileal CD mucosal *E. coli* strain, LF82 [2]. Here we have shown that all CD mucosa-associated *E. coli* tested (whether isolates were ileal, ileal colonic or colonic in location, disease site and/or activity) were able to grow at a low pH. Key CD mucosa-associated strains shown to be adherent and invasive to epithelial cells, including LF82 and HM605, were also shown in this study to grow and survive in a nutrient-depleted (M9 minimal media) environment. This supports earlier data that intra-macrophage survival and replication of the paradigm ileal CD *E. coli* isolate LF82 was dependent on an acidic environment [47]. A number of key bacterial stress response proteins have been implicated to support survival and persistence of LF82 within macrophages, such as the bacterial chaperone and serine protease high temperature requirement A (HtrA) and the bacterial thiol:disulfide bond oxidoreductase DsbA [47,48]. However, genes encoding these two bacterial enzymes were seen to be ubiquitous in an extensive screen of 281 colonic mucosa-associated isolates, whether obtained from patients with CD, UC, CRC or non-inflamed control patients [38]. Overall, the ability to persist and replicate within the low-pH environment of the macrophage vacuoles, suggests that alkalinisation of the intra-phagolysosome would perhaps be a good approach to target and reduce CD *E. coli* survival within macrophages, and subsequently to attenuate mucosal inflammation. Hydroxychloroquine, a weak base with the ability to increase phagolysosome pH, has been shown to improve intra-macrophage killing of bacteria with an intra-phagolysosome life-style, such as *Coxiella burnetii*, causing Q-fever [49] and *Tropheryma whipplei*, causing Whipple’s disease [50,51]. We have recently shown that hydroxychloroquine, at concentrations achievable in vivo, reduces survival and replication of intra-macrophage CD mucosal *E. coli* [25]. Hydroxychloroquine also had marked synergistic effects with antibiotics that were seen to be effective against intracellular *E. coli* [14,25]. With this in mind, we are currently undertaking a trial of combination antibiotics and hydroxychloroquine (APRiCCOT—ClinicalTrials.gov Identifier: NCT01783106) [52]. Other approaches to enhance CD mucosa-associated *E. coli* intra-macrophage killing could include the use of vitamin D supplementation [25]. 

Macrophage function and clearance of bacterial infection is not altered by the absence of Nfκb1 p50 subunit in vivo [53]; however, here we have shown that exemplar CD mucosal *E. coli* strains LF82 and HM605 cannot survive within *Nfκb1^-/-^* BMDMs, suggesting that inhibiting classical NF-κB pathway signalling specifically within macrophages could be therapeutically useful [54]. NF-κB signalling involves actions of five family member protein subunits/protein subunit complexes, including NF-κB1, NF-κB2, RelA (p65), RelB and c-Rel, controlling DNA transcription and subsequent expression of pro-inflammatory cytokines (such as TNF) to play a pivotal role in regulating immune response to infection [54,55]. High levels of TNF secreted by J774.A1 macrophages harbouring CD mucosa-associated *E. coli* strains, such as LF82 and 13I, are thought to support intra-macrophage survival and replication [31,32], with exogenous addition of TNF shown also to increase intra-macrophage persistence of *E. coli* LF82 [32]. Survival of these particular CD *E. coli* strains within murine macrophage phagolysosomes appears to involve initial suppression of acute NF-κB signal pathway activation within the early phase of infection [31], a common strategy used by other pathogenic bacteria to support intra-cellular survival [56,57]. Persistence of CD mucosal *E. coli* during the later phase of infection, however, likely results in a chronic activation of NF-κB, correlating to increased release of TNF observed from infected macrophages [31]. 

Various anti-inflammatory and immunosuppressant agents, including glucocorticoids, strongly inhibit NF-κB activation by mechanisms that are not fully understood [53]. The evidence reported here, that pre-treatment of macrophages with hydrocortisone (at therapeutic doses), is perhaps surprising but has been reported previously for other corticosteroids [58,59], although there are also reports of a lack of impact of corticosteroids on bacterial killing by macrophages or neutrophils [60,61]. Although hydrocortisone exerts its effect mainly by acting on mucosal immune cells such as T cells, monocytes, macrophages and dendritic cells [62], we have shown that hydrocortisone also blocks NF-κB activated pro-inflammatory cytokine release from intestinal epithelial cells following infection with IBD mucosa-associated *E. coli* and is also beneficial in enhancing mucosal barrier function [62,63]. There is of course no doubt that corticosteroid therapy increases the risk of sepsis in many situations, including CD [64], and it is probable that other actions of corticosteroids including impairment of leucocyte chemotaxis contribute to this [65]. 

The studies reported here suggest that a macrophage-targeted inhibition of NF-κB activation could be a plausible therapeutic strategy for CD. Despite considerable advances over the last decade or so, the role in CD of *E. coli* lacking conventional pathogenicity remains intriguing but unproven until it can be shown that therapeutic actions targeting the *E. coli* improve the condition.

## 4. Materials and Methods 

### 4.1. Murine Bone Marrow Isolation

Ten- to 12-week-old wild-type C57BL/6 (Charles River, Margate, UK) and *Nfκb1^-/-^* mice, bred on the C57BL/6 genetic background [66], were maintained at the University of Liverpool’s specific pathogen-free (SPF) Biomedical Services Unit under a 12:12 hour light/dark cycle and fed a standard pelleted chow diet. All mice were euthanized by cervical dislocation following UK Home Office Animals Scientific Procedures Act 1986 [67]. Bone marrow progenitor cells were obtained from femurs of four C57BL/6 mice (*n* = 2 male, *n* = 2 female) and 4 *Nfκb1^-/-^* transgenic mice (*n* = 1 male, *n* = 3 female). Briefly, femurs from each mouse were flushed with culture medium and progenitor cells for each mouse were independently differentiated to macrophages with murine macrophage colony-stimulating factor (M-CSF) as previously described [68]. Maturation of BMDMs in culture was monitored by immunocytochemistry using F4/80/EMR1 primary antibody (CI-A3-1, Novus Europe, Abingdon, UK), with secondary anti-mouse Ig antibody/diaminobenzidine (DAB) substrate detection (Vector Labs, Peterborough, UK). No differences were observed in growth, differentiation (following 6 d treatment with M-CSF) and maturation (F4/80 positivity) of *Nfκb1^-/-^* BMDMs compared to C57Bl/6 BMDMs.

### 4.2. Bacteria and Culture Conditions 

Bacteria stored at ‒80 °C using the Protect™ bacteria preservation system (Fisher Scientific, Loughborough, UK) were sub-cultured overnight at 37 °C on Luria‒Bertani (LB) solid agar plates prior to use in experimental assays. 

#### 4.2.1. CD *E. coli* Strains

Mucosa-associated *E. coli* strains LF10, LF11, LF13, LF82 and LF86, were previously isolated from inflamed lesions of clinically active ileal CD patients [1,2,69]. *E. coli* strain 541-15A was previously isolated from patient with CD involving the ileum [4,35]. CD mucosa-associated strains HM95, HM96, HM104, HM413 and HM419 were isolated from non-inflamed colonic mucosa of patients in remission but with a history of active ileal inflammation. Isolate HM427 was obtained from the non-inflamed colon tissue of a CD patient who previously had ileo-colonic inflammation. HM154, HM580, HM605 and HM615 were isolated from inflamed colon biopsy tissue of CD patients [3].

#### 4.2.2. UC *E. coli* Strains

Five strains, HM233, HM250, HM378, HM394 and HM457, were previously isolated from inflamed colon biopsy tissue of clinically active UC patients, with a further strain, HM464, having been isolated from the colonic mucosa of a UC patient in remission [3].

#### 4.2.3. CRC *E. coli* Strains

Seven strains, HM44, HM229, HM230, HM244, HM312, HM358 and HM374, were previously isolated from the colonic mucosa of patients with CRC [3]. 

#### 4.2.4. Other *E. coli* Strains

Additional reference *E. coli* strains were obtained from the STEC Centre (Department of Microbiology and Molecular Genetics, Michigan State University, East Lansing, MI, USA [70]), including ECOR40 and ECOR50 (isolated from patients with acute pyelonephritis), ECOR48 and ECOR64 (isolates from patients with acute cystitis). Uropathogenic *E. coli* (UPEC) included SJH2 and J96 (obtained from existing cryopreserved cultures within the Department of Clinical Infection, University of Liverpool, UK) and CP9 [71]. *E. coli* strains obtained from healthy individuals included ECOR1, ECOR35 and ECOR51 (STEC Centre). Other mucosally associated strains were isolated previously from the colon of otherwise healthy individuals with irritable bowel syndrome (HM484 and HM488), sporadic polyps (HM428, HM454 and HM456) or with haemorrhoids (HM463) [3]. *E. coli* K-12 (*E. coli* (Migula) Castellani and Chalmers ATCC^®^ 10798) was obtained from the American Type Culture Collection (LGC Standards, Middlesex, UK), *E. coli* XL-1Blue from Agilent Technologies (Santa Clara, CA, USA) and *E. coli* K-12 derivative EPI300-T1 from Epicentre (Madison, WI, USA). The latter strain was used as a negative control in the intra-macrophage replication assays.

### 4.3. Bacteria Stress Tolerance Tests 

Stress tolerance tests were carried out as per [72]. Briefly, bacterial cultures were grown at 37 °C in LB medium to an OD_600__nm_ 0.1, and diluted in 10-fold serial dilution steps in sterile physiological saline. Aliquots (20 µL) from each dilution were spotted, in triplicate, to LB agar plates under the following stress conditions; LB agar pH 7.0 alone (standard conditions); LB agar containing 100 mM 4-morpholine ethanesulfonic acid (MES) pH 5.0 (low pH), with or without 1 mM sodium nitrite (low pH and nitrosative stress); 1 mM hydrogen peroxide, pH 7.0 (oxidative stress); or 1 mM methyl viologen, pH 7.0 (superoxide stress). Plates were incubated overnight at 37 °C. All chemicals were purchased from Sigma (Poole, UK).

### 4.4. Bacteria Survival and Growth in Acidic Nutrient-poor M9 Medium

At early exponential growth phase (OD_600nm_ = 0.1), bacteria were re-suspended in M9 minimal salts microbial growth medium (Life Technologies Ltd, Paisley, UK) supplemented with 0.1% w/v Casamino Acids (MP Biomedicals, Loughborough, UK), 100 mM Bis-Tris (Sigma), 0.16% v/v glycerol (Sigma) and 10 μM magnesium chloride (Sigma), both at pH 7.0 and pH 4.5. OD of each bacterial suspension was measured on a spectrophotometer hourly up to 8 h.

### 4.5. Intra-Macrophage Replication Assays

Murine macrophage-like cell line J774-A1, obtained from the European Collection of Animal Cell Culture (ECACC #91051511; Porton Down, Salisbury, UK [73]), was maintained in RPMI 1640 medium (Sigma) supplemented with 10% v/v foetal calf serum (Life Technologies, Paisley, Scotland), 100 U/mL penicillin (Sigma), 100 µg/mL streptomycin (Sigma), and 4mM L-glutamine (Sigma), within 75-cm^2^ tissue culture flasks (Appleton Woods Limited, Birmingham, UK). All macrophages (BMDM and J774.A1) were seeded onto 24-well tissue culture plates at a density of 1 x 10^5^ cells per well. The ability of murine BMDM and J774A.1 macrophages to kill phagocytosed bacteria was assessed by a gentamicin protection assay previously described [14,25]. Briefly, following a 2 h incubation at 37 °C to allow internalization of bacteria (multiplicity of infection, MOI 10), cell monolayers were washed thrice with sterile PBS to remove non-adherent bacteria and treated with fresh culture medium containing 20 µg/mL gentamicin for 1 h to kill extracellular bacteria. Following this, cells were washed with sterile PBS and replaced with a fresh medium containing 20 µg/mL gentamicin and incubated for a further 3 h at 37 °C. Data were expressed as relative fold change of recovered intra-macrophage bacteria at the end of the further 3-h incubation period (6 h post-infection) compared to the number of viable colony forming units (cfu) obtained immediately after the 1 h gentamicin treatment step (i.e., 3 h post-infection).

### 4.6. Effect of Hydrocortisone Pre-Treatment of Macrophages on Intracellular Replication of *E. coli*

J774-A1 murine macrophages were pre-incubated for 24 h in the presence of hydrocortisone (0.06 to 6 µM) and were then infected with CD *E. coli* HM605 (MOI 10) and killing/replication assessed as per [14]. Assessment of hydrocortisone treatment on J774-A1 macrophage cell viability was performed using the Toxilight assay (Lonza, Slough, UK) to assess for cytotoxicity, following release of adenylate kinase to culture medium. Hydrocortisone at concentrations tested was also assessed for any direct effect on *E. coli* growth in the absence of macrophages, assessed by enumeration of bacteria colony-forming units (CFU) following overnight growth on standard LB agar.

### 4.7. Data Analysis 

Statistical comparisons of normally distributed datasets were performed by one-way analysis of variance (ANOVA) followed by Dunnett’s post hoc test for pair-wise comparisons between treated and untreated/uninfected controls. Where there was evidence of non-normality or lack of homogeneity of datasets, the data were rank-transformed and non-parametric Kruskal–Wallis one-way ANOVA was used (StatsDirect version 2.6.2, Sale, UK). Differences were considered significant when *p* < 0.05. Non-parametric correlation coefficient (Spearman’s rank) analysis was used to assess for any association between % bacterial growth in stress conditions and the ability to replicate inside murine macrophages. Visualisation of stress response data of *E. coli* isolates was performed using expression heat map freeware Heatmapper (University of Alberta, Edmonton, AB T6G 2E8, Canada) [74].
